# A microbial eukaryote with a unique combination of purple bacteria and green algae as endosymbionts

**DOI:** 10.1126/sciadv.abg4102

**Published:** 2021-06-11

**Authors:** Sergio A. Muñoz-Gómez, Martin Kreutz, Sebastian Hess

**Affiliations:** 1 Institute for Zoology, Cologne Biocenter, University of Cologne, Zülpicher Str. 47b, 50674 Cologne, Germany.; 2 Center for Mechanism of Evolution, The Biodesign Institute, School of Life Sciences, Arizona State University, 727 E. Tyler St., Tempe, AZ 85281-5001, USA.; 3 Private Laboratory, Am See 27, 78465 Constance, Germany.

## Abstract

Oxygenic photosynthesizers (cyanobacteria and eukaryotic algae) have repeatedly become endosymbionts throughout evolution. In contrast, anoxygenic photosynthesizers (e.g., purple bacteria) are exceedingly rare as intracellular symbionts. Here, we report on the morphology, ultrastructure, lifestyle, and metagenome of the only “purple-green” eukaryote known. The ciliate *Pseudoblepharisma tenue* harbors green algae and hundreds of genetically reduced purple bacteria. The latter represent a new candidate species of the Chromatiaceae that lost known genes for sulfur dissimilation. The tripartite consortium is physiologically complex because of the versatile energy metabolism of each partner but appears to be ecologically specialized as it prefers hypoxic sediments. The emergent niche of this complex symbiosis is predicted to be a partial overlap of each partners’ niches and may be largely defined by anoxygenic photosynthesis and possibly phagotrophy. This purple-green ciliate thus represents an extraordinary example of how symbiosis merges disparate physiologies and allows emergent consortia to create novel ecological niches.

## Teaser

A eukaryote with two contrasting intracellular photosymbionts has a unique combination of physiological properties.

## INTRODUCTION

Symbioses between heterotrophs and photosynthetic algae (photosymbiosis or acquired phototrophy) are widespread and ecologically important in today’s ecosystems (*1*–*4*). Many eukaryotes have thus become mixotrophs, i.e., they combine predation and photosynthesis, by acquiring algal endosymbionts or chloroplasts from algae. Photoautotrophic endosymbionts often release photosynthetic products (e.g., sugars, organic acids, and oxygen) into their hosts, whereas hosts provide a nutrient-rich environment (e.g., nitrogen and minerals) and physical protection against predators and viruses (*5*–*7*). Today, virtually all known photosymbioses involve cyanobacteria or eukaryotic algae that perform oxygenic photosynthesis (*1*, *2*), except for the marine ciliate *Strombidium purpureum*, whose purple bacterial endosymbionts remain uncharacterized (see Supplementary Text for details) (*8*, *9*). Only one eukaryote has ever been described as harboring both green algae and purple bacteria in the same host cell (see below) (*10*, *11*). This is a puzzling combination as these two groups of photosynthesizers typically occupy different niches in nature. Green algae are aerobic and oxygenic, whereas purple bacteria are primarily anaerobic and anoxygenic (*12*). Moreover, green algae and purple bacteria absorb distinct wavebands because of the different photosynthetic pigments [e.g., the absorption peak of chlorophyll *a* is at ~600 to 700 nm and those of bacteriochlorophyll *a* at ~800 to 900 nm; (*13*, *14*)]. It is thus unknown how two endosymbionts with these contrasting photosynthetic physiologies came to coexist in the same host cytoplasm and how this complex symbiotic consortium makes a living in its environment.

## RESULTS AND DISCUSSION

### *Pseudoblepharisma tenue* is a phagotroph with two photosynthetic endosymbionts

**Fig. 1:**
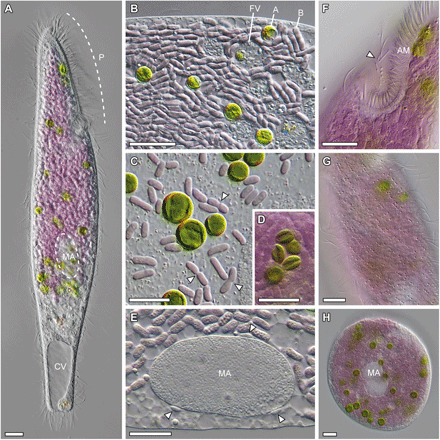
Morphology of *P. tenue* and its two photosynthetic endosymbionts. (**A**) A motile individual of *P. tenue* displaying a peristome (P) that occupies the first third of the cell, a posterior contractile vacuole (CV), and an elliptic macronucleus (MA) largely obscured by the photosynthetic endosymbionts. (**B**) Detail of *P. tenue* cytoplasm with purple bacteria (B), green algae (A), and food vacuoles (FV) with partially digested material. (**C**) Purple bacteria and green algae in a squashed ciliate. Note dividing bacteria (arrowheads). (**D**) Ciliate cytoplasm with small, elliptic algal cells likely originating from autospore formation. (**E**) Single elliptic macronucleus (MA) with peripheral micronuclei (arrowheads). (**F**) Peristome with adoral membranelles (AM) and opposing row of ciliated dikinetids (arrowhead). (**G**) Cortex of *P. tenue* with slightly helical rows of refractive, colorless granules (putative mucocysts). (**H**) Cyst-like cell of *P. tenue* with endosymbionts and two macronuclei (MA). Scale bars, 10 μm. See also movie S1 and fig. S2 for ultrastructure.

We found populations of a purple-green ciliate dwelling in the *Sphagnum* ponds of the Simmelried moorland (Constance, Germany), a “hotspot” for microbial diversity (Fig. 1, fig. S1, and movie S1) (*15*). This ciliate most closely resembled *P. tenue* KAHL, which was briefly described in 1926 as a very uncommon ciliate (*10*) and since then never studied in detail (*11*, *16*). The characteristic purple-green color of *P. tenue* is conferred by two different endosymbionts: purple bacteria and green algae (Fig. 1, A to C, and fig. S2, A to F). The presence of purple bacteria and green algae dividing in the cytoplasm of *P. tenue* suggests that both endosymbionts are inherited in a vertical fashion (Fig. 1, C and D). Moreover, food vacuoles with partially digested contents, which clearly differ from degraded endosymbionts, also suggest that *P. tenue* is capable of phagocytosis (Fig. 1B). The major morphological features of this ciliate—such as cell body shape and length (182.5 μm on average, *n* = 6; table S1), posterior contractile vacuole (Fig. 1A), macro- and micronuclei (Fig. 1E), peristome and associated structures (Fig. 1F), and cortical granules (putative mucocysts; Fig. 1G)—all agree with the original description of *P. tenue* (*10*, *11*) and suggest an affiliation to the class Heterotrichea (Ciliophora). Unlike *Blepharisma* species, *P. tenue* lacks a conspicuous flap-like undulating membrane alongside the peristome and thus rather resembles *Spirostomum* species (Fig. 1F); however, *Spirostomum* species are often larger and more contractile than *P. tenue*. In addition, we also observed spherical cells of *P. tenue* that potentially are thin-walled resting cysts (Fig. 1H).

### *P. tenue* is a heterotrich ciliate inhabited by a known *Chlorella* strain and a new purple sulfur bacterium

**Fig. 2:**
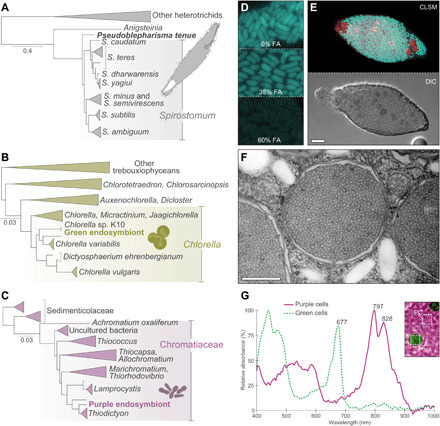
Phylogenetic identity of the symbiotic partners, cellular localization of the endosymbionts, and details of “*Ca.* Thiodictyon intracellulare”. (**A**) Maximum likelihood phylogenetic tree of the Heterotrichea based on the concatenated nuclear rRNA gene operon (including sequences of 18*S* rRNA, ITS1, 5.8*S* rRNA, ITS2, and D1D2-28*S* rRNA regions) and the mitochondrial *COI* gene. *P. tenue* is sister to *Spirostomum* species with moderate support. (**B**) Maximum likelihood phylogenetic tree of the Chlorellaceae based on the chloroplast-encoded *rbcL* gene. The green algal endosymbionts of *P. tenue* are identical in their *rbcL* gene sequence to *Chlorella* sp. K10, an endosymbiont of *H. viridissima*. (**C**) Maximum likelihood tree of the Chromatiaceae based on the 16*S* rRNA gene. The purple bacterial endosymbionts are most closely related to *Thiodictyon* species. (**D**) Fluorescence signal of probe Thio643-Cy3 in spread-out bacterial cells from *P. tenue* cytoplasm. Probe-conferred fluorescence (shown in pseudocolor) decreases with increasing formamide (FA) concentration (expected binding behavior). (**E**) Confocal fluorescent (CLSM) and transmitted light [DIC (differential interference contrast)] micrographs of a *P. tenue* cell displaying the FISH signal for probe Thio643-Cy3 (cyan; pseudocolor) and the autofluorescence of green algal chlorophyll (red; pseudocolor). See also movie S2. (**F**) Transmission electron micrograph of “*Ca.* Thiodictyon intracellulare” and starch granules in the cytoplasm of *P. tenue*. The bacterial cytoplasm is packed with vesicular chromatophores and surrounded by three membranes (see also fig. S2, D to F). (**G**) Absorbance spectra of purple and green endosymbionts recorded with hyperspectral microscopy. Note the peaks in the infrared range (>700 nm) typical for bacteriochlorophyll. Negative absorbance values in the region >900 nm are artifacts probably based on a low signal-to-noise ratio. Representative sample areas for purple cells (PC) and green cells (GC) are shown in the inset (for details, see fig. S4). Scale bars, 10 μm (E) and 100 nm (F).

To determine the phylogenetic affiliation of each symbiotic partner, we relied on a single-cell polymerase chain reaction (PCR) approach; attempts to establish cultures of *P. tenue* and its endosymbionts were unsuccessful. Phylogenetic analyses of the ribosomal RNA (rRNA) gene operon and the mitochondrial *COI* gene revealed that *P. tenue* is most closely related to ciliates of the genus *Spirostomum* (Fig. 2A and fig. S3A). All *Spirostomum* species are purely heterotrophic predators, except for *Spirostomum semivirescens*, which harbors zoochlorellae (*17*–*19*); *P. tenue*, however, was phylogenetically distant from *S. semivirescens* in our phylogenetic trees (Fig. 2A and fig. S3A). Although *P. tenue* has a clear affinity to *Spirostomum*, it remains to be determined whether it is sister to the genus *Spirostomum* or represents a new lineage within it (Fig. 2A and fig. S3A).

The algal endosymbionts are most closely related to *Chlorella* species (Trebouxiophyceae) according to phylogenetic analysis of the 18*S* rRNA-ITS2 and *rbcL* genes (Fig. 2B and fig. S3B). This is consistent with the fact that many eukaryotes harbor *Chlorella*-like endosymbionts (i.e., “zoochlorellae”) (*2*). Unlike most *Chlorella* species (*20*), the green algal endosymbionts did not have visible pyrenoids under the light microscope (Fig. 1C), but a pyrenoid-like structure was clear in our electron micrographs (fig. S2, B and C). The *rbcL* gene of the green endosymbionts is identical to that of *Chlorella* sp. K10, an endosymbiont of the freshwater cnidarian *Hydra viridissima* strain K10 isolated in Switzerland (Fig. 2B and fig. S3B) (*21*). Because *H. viridissima* has co-speciated with its *Chlorella* endosymbionts (*21*), it is possible that the ancestor of *P. tenue* acquired *Chlorella* sp. K10 after it was released from a cnidarian host.

The bacterial endosymbionts are most closely related to the genus *Thiodictyon* (Chromatiaceae, Gammaproteobacteria) according to phylogenetic analyses of their 16*S* rRNA gene (Fig. 2C and fig. S3C). We confirmed the localization of the *Thiodictyon*-related bacteria in the cytoplasm of *P. tenue* by fluorescence in situ hybridization (FISH) (Fig. 2, D and E, and movie S2; see Materials and Methods for details). Most members of the Chromatiaceae, known as “purple sulfur bacteria,” are anaerobic and anoxygenic photosynthesizers that often use hydrogen sulfide as a photosynthetic electron donor and accumulate sulfur globules in their periplasm (*22*). The conspicuous purple-violet color of the bacterial endosymbionts (Fig. 1, B and C) and the abundant vesicular “chromatophores” (Fig. 2F and fig. S2, E and F) agree with them being a member of the Chromatiaceae (*22*). Absorbance spectra of the purple endosymbionts by hyperspectral microscopy reveal clear peaks in the infrared spectrum (Fig. 2G and fig. S4B) that are typical for bacteriochlorophyll-containing organisms (*12*). The *Thiodictyon*-related endosymbionts do not appear to accumulate highly refractile sulfur globules (Figs. 1, C and E, and 2F), and we also did not recover discrete sulfur signals through scanning electron microscopy–energy-dispersive x-ray spectroscopy. This suggests that the photosynthetic physiology of the *Thiodictyon*-related endosymbiont might have diverged from that of its closest free-living relatives. Moreover, the *Thiodictyon*-related endosymbionts are of great interest as no other photosynthetic purple sulfur bacteria are known to be endosymbiotic (*22*); there are, however, several nonphotosynthetic, sulfur-oxidizing endosymbionts (e.g., “*Candidatus Thiosymbion*”) (*23*). On the basis of its phylogenetic placement as sister to known *Thiodictyon* species (Fig. 2C) and the 0.91 average nucleotide identity to its closest genome-sequenced relative (see Materials and Methods), we propose a new candidate species for the purple bacterial endosymbiont of *P. tenue* [in accordance with published recommendations (*24*)]:

“*Candidatus* Thiodictyon intracellulare” [(Chromatiales, Gammaproteobacteria) NC; NA; R; NAS (GenBank number MW203126), oligonucleotide sequence complementary to unique region of 16*S* rRNA is 5′-CTC TCA GAC TCT AGC GCG TCA-3′ (probe Thio643); S (*P. tenue*, Ciliophora; cytoplasm); probably microaer., reduced metabolism (probably no sulfur dissimilation); M]. Muñoz-Gómez, Kreutz, and Hess, *Sci. Adv.*, **7**: eabg4102, 2021. Etymology: intrā (Latin) = inside, cellula, -ae f (diminutive of cella) (Latin) = small room, cell. The epithet “intracellulare” refers to the intracellular lifestyle of the candidate species. Additional information: Rod-shaped, sometimes slightly curved, purplish cells colonizing the cytoplasm of *P. tenue* (Heterotrichea, Ciliophora) in large numbers (about 900 to 1300 bacterial cells per host cell); vegetative cells about 6 × 2 μm on average, bounded by two membranes and an additional vacuolar membrane, and packed with vesicular chromatophores discernible in transmission electron microscopy (TEM) micrographs; no other life history stages observed; genetically defined by the 16*S* rRNA gene sequence MW203126 and the draft genome JADNEJ000000000 (both GenBank). Reference material: Resin block for TEM (NHMUK 2021.4.15.1; deposited in the “Protists Collection” at the Department of Life Sciences of the National History Museum in London (Cromwell Road, UK) containing a single host cell collected in pond II of the “Simmelried” moorland, Constance, Germany; 47.717767, 9.09375.

### “*Ca.* Thiodictyon intracellulare” has a reduced and specialized metabolism

To gain further insights into the possible metabolic interactions between *P. tenue* and its photosynthetic endosymbionts, we sequenced the metagenome of this tripartite symbiotic consortium. The metagenome was assembled from three deeply sequenced short-read libraries made from amplified genomes of three separately isolated single cells (see Materials and Methods for details). The metagenomic scaffolds mostly group into three main clusters that correspond to nearly complete genomes for each symbiotic partner (fig. S5). The genome of “*Ca.* T. intracellulare” is less than half the size of that of its closest known relative, “*Candidatus* Thiodictyon syntrophicum” [2.9 versus 7.74 million base pairs (Mbp); 3141 versus 6771 genes; table S2] (*25*). Such genome reduction is consistent with an endosymbiotic lifestyle (*26*) and has been accompanied by extensive gene loss and pseudogenization (figs. S6 and S7 and table S2).

**Fig. 3:**
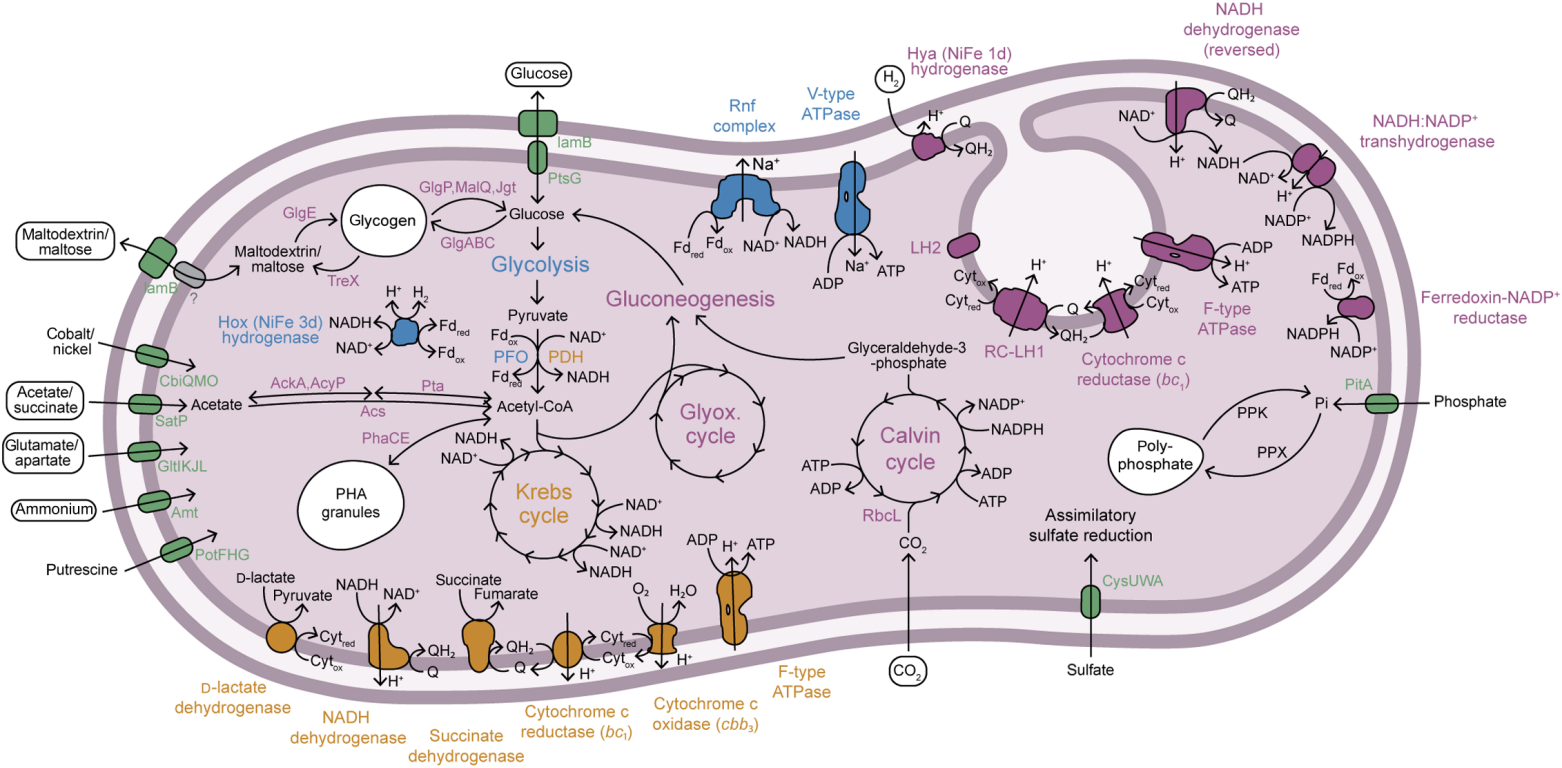
Predicted metabolism of the purple endosymbiont “*Ca.* T. intracellulare”. Inferred physiology and energy metabolism of the purple endosymbiont “*Ca.* T. intracellulare”. Protein complexes that are part or contribute to the photosynthetic electron transport chain (purple), micro-aerobic respiration (orange), and fermentation (blue). Metabolite transporters are shown in green. Sources of nitrogen and carbon/electrons and potential photosynthate are shown in rounded rectangles.

“*Ca.* T. intracellulare” lost genes for major metabolic pathways such as the capacity to use reduced sulfur compounds as electron donors for photosynthesis (sulfur dissimilation), a hallmark of purple sulfur bacteria (*27*). On the basis of the absence or pseudogenization of several important genes, “*Ca.* T. intracellulare” lost the capacity to oxidize sulfide (e.g., *sqrD* and *sqrF* genes), elemental sulfur (e.g., *dsrA* and *dsrB* genes), and thiosulfate (e.g., *soxY* and *soxZ*) (data S1). These predicted losses are consistent with the absence of visible sulfur globules in the purple endosymbionts (Figs. 1, C and E, and 2F). Furthermore, the capacity to fix atmospheric dinitrogen (e.g., *nifD* and *nifK*) and hydrolyze urea (e.g., *ureA*, *ureB*, and *ureC*) as well as the ability to store excess nitrogen as cyanophycin (e.g., *cphA*) were also lost by “*Ca.* T. intracellulare” (data S1). However, “*Ca.* T. intracellulare” might be able to acquire nitrogen from its host because it retained transporters for glutamate and/or aspartate and ammonium (Fig. 3 and data S1). Moreover, “*Ca.* T. intracellulare” retained many genes involved in pathways for carbon and energy metabolism, such as photosynthesis (e.g., photosystem and light-harvesting antennae), carbon fixation [RuBisCO (ribulose-1,5-bisphosphate carboxylase-oxygenase) and the Calvin cycle], respiration under hypoxic conditions (e.g., cytochrome *cbb*_3_), fermentation (e.g., pyruvate:ferredoxin oxidoreductase and Hox [NiFe] hydrogenase), and the capacity to store excess carbon as glycogen and polyhydroxyalkanoate (PHA) granules (see Fig. 3 and data S1). Furthermore, “*Ca.* T. intracellulare” has transporters for small organic acids (i.e., acetate and succinate), glucose, maltose, and phosphate, which might be imported from the host cytoplasm (see Fig. 3, data S1, and Supplementary Text). Similar to *Allochromatium vinosum*, free-living *Thiodictyon* species are metabolic generalists that can use both reduced inorganic and organic compounds as electron donors (*22*, *25*, *28*). Therefore, the reduced and more specialized metabolism of “*Ca.* T. intracellulare” is likely to be the outcome of an endosymbiotic lifestyle in a nutrient-rich environment.

### The ciliate host and the green algal endosymbiont genomes harbor genes for fermentation

The endosymbiotic *Chlorella* sp. most probably has the capacity to photosynthesize oxygenically and respire aerobically as its free-living relatives do. However, this endosymbiotic green alga may also be able to ferment both in the light and in the dark (*29*, *30*). *Chlorella* sp. has retained multiple fermentative pathways in the cytosol, mitochondrion, and chloroplast that could result in the excretion of multiple fermentative end products, including d-lactate, acetate, ethanol, and molecular hydrogen (fig. S8A, Supplementary Text, and data S2). The *P. tenue* ciliate host has typical aerobic mitochondria, as evidenced by its conspicuous mitochondrial cristae (fig. S2, G and H) and the retention of a conventional ciliate mitochondrial genome (Figshare DOI: 10.6084/m9.figshare.13140560). However, the ciliate host can potentially carry out mitochondrial fermentation by using fumarate as an electron acceptor via a reversed complex II and rhodoquinone. This type of fermentation leads to the excretion of acetate and propionate as end products (fig. S8B, Supplementary Text, and data S3).

### *P. tenue* lives as a mixotroph by means of phagotrophy and anoxygenic photosynthesis

**Fig. 4:**
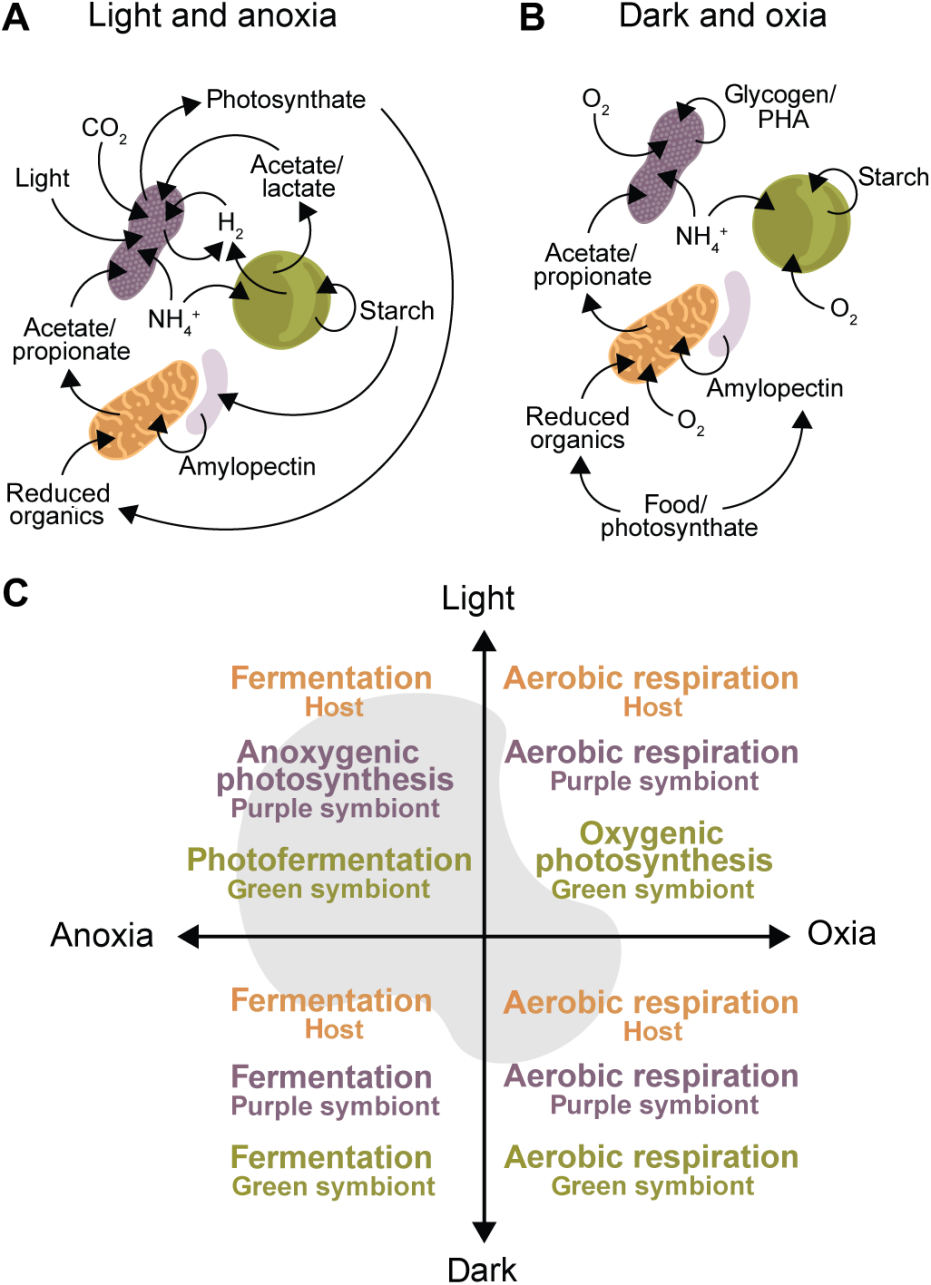
Potential metabolite exchange among partners and predicted preferred physiology of each partner in four discrete microenvironments. (**A**) Most plausible metabolic interactions, as inferred from genome data, under light and hypoxic/anoxic conditions. (**B**) Most plausible metabolic interactions, as inferred from genome data, under dark and micro-oxic conditions. (**C**) Major physiological modes of the *P. tenue* symbiotic consortium across two environmental variables, light intensity and oxygen concentration, as inferred based on genome data and lifestyle of closest free-living relatives. In addition to light-anoxia and dark-oxia, two other general physiological modes are possible by this symbiotic consortium. In light-oxia, all three partners respire aerobically, but the green algae also photosynthesize oxygenically; the chloroplasts of the green algal endosymbionts of *P. tenue* accumulate starch that suggests that they actively photosynthesize (see Supplementary Text). In dark-anoxia, the whole symbiotic consortium turns to fermentation of ingested food or stored carbon (*36*). Nevertheless, intermediate metabolic states between these four major modes might occur. See also fig. S8.

*P. tenue* represents a physiologically complex and potentially flexible symbiotic consortium. This is because each symbiotic partner has more than one type of energy metabolism or mode of growth (Fig. 4, A to C). The physiological mode preferred by each symbiotic partner may be primarily determined by two major environmental factors: light and oxygen (Fig. 4C and fig. S9). Despite the aforementioned metabolic complexity, which suggests ecological flexibility, the *P. tenue* symbiotic consortium appears to occupy a rather narrow niche in its environment. This is suggested by three observations. First, *P. tenue* preferentially dwells in hypoxic sediments rich in organic matter, where it phagocytoses and digests bacteria as food (see Fig. 1B for evidence of digestive vacuoles); this lifestyle is similar to that of *Spirostomum* species that are voracious bacterivores and often venture into hypoxic microenvironments to find their prey (*18*, *31*, *32*). Second, *P. tenue* co-occurs with diverse free-living purple sulfur bacteria (fig. S1). The plant-covered and organic matter–rich sediments where *P. tenue* is found presumably filter far-red and near-infrared light (*13*) and offer plenty of organic compounds and sulfide that select for metabolically versatile purple bacteria like the putative ancestor of “*Ca.* T. intracellulare” (*22*, *25*). Dwelling in hypoxic sediments might therefore favor anoxygenic photosynthesis by the purple symbionts over oxygenic photosynthesis by the green algal symbionts. Third, most of the volume of the *P. tenue* symbiotic consortium is occupied by “*Ca.* T. intracellulare” (Fig. 1A). The purple endosymbionts represent about 26.4% of the volume of the whole symbiotic consortium, whereas the green endosymbionts only represent about 1.4% (table S1 and Supplementary Text). Together, these observations suggest that the physiology of the symbiotic consortium largely follows that of its purple endosymbionts, which may primarily photosynthesize anaerobically (e.g., “*Ca.* T. intracellulare” synthesizes bacteriochlorophyll anaerobically through an oxygen-independent BchE enzyme). We conclude that *P. tenue* is a mixotroph with a dual lifestyle that combines anoxygenic photosynthesis in the light and micro-aerobic phagotrophy (Fig. 4, A to C, and fig. S9).

### What metabolic interactions might underlie the *P. tenue* symbiosis

On the basis of our microscopic observations and inferences from metagenomic data, the following metabolic interactions among the symbiotic partners in *P. tenue* are most plausible. In the light and the absence of oxygen, the purple endosymbionts most probably photosynthesize anoxygenically, while both the ciliate host and the green endosymbionts ferment reduced organic compounds (Fig. 4, A and C); green algae such as *Chlamydomonas reinhardtii* or the salamander-symbiont *Oophila amblystomatis* switch to hydrogen-producing fermentation in hypoxia and light [(*33*) and references therein] (it is also possible, in principle, that the green algae photosynthesize under hypoxia, and the oxygen released is quickly consumed by host mitochondria). The purple symbionts can use either hydrogen or organics as electron donors. Hydrogen might be released by fermentation from the green alga (by means of its [FeFe] hydrogenase) or by photo-fermentation from the purple symbiont itself (through an [NiFe] hydrogenase) (Figs. 3 and 4A). Organic compounds are produced by both algal (e.g., acetate and lactate) and host mitochondrial (acetate and propionate) fermentation (Fig. 4A and fig. S8). The purple symbionts have, indeed, retained transporters for these small organic acids (i.e., acetate or succinate) and for glucose (see above; Fig. 3). The energy derived from the anoxygenic photosynthetic chain of the purple symbionts is likely used to fix inorganic carbon (CO_2_) derived from the host metabolism and the environment. The fixed carbon can then be stored as glycogen or PHA granules (rarely seen in electron micrographs) or, more likely, released and stored in the host as starch (Fig. 4A). The latter is suggested by the presence of numerous electron-translucent granules in the cytoplasm of *P. tenue* [presumably amylopectin (*34*); fig. S2, E and F]. We expect that “*Ca.* T. intracellulare” supplies photosynthate in the form of small organic compounds to its host, as known from many other photosynthetic endosymbionts (*7*, *35*–*38*). As a nitrogen source, the purple symbionts (as well as the green algal symbionts) might import ammonium and glutamate/aspartate from the ciliate host (Fig. 3; see above) as is also seen in many other photosymbioses (*36*, *39*–*42*). This agrees with the observation that “*Ca.* T. intracellulare” lost the capacity to store fixed nitrogen but retained ammonium and glutamate/aspartate transporters (Fig. 3).

In the dark, all symbiotic partners probably respire aerobically to fully oxidize reduced organic compounds, which derive from stored carbon (e.g., starch for the host and green alga and glycogen or PHA for the purple symbiont; Fig. 4B) or phagocytosed food (Figs. 1B and 4B). This is expected because each symbiotic partner has micro-aerobic respiratory chains (Fig. 3 and fig. S8), and respiration is much more efficient than fermentation. Then, it might well be that the *P. tenue* symbiotic consortium moves into slightly more oxygenated microenvironments to respire micro-aerobically in the dark. Vertical migration in response to oxygen concentration is indeed known from various ciliates, including *Spirostomum* species (*43*, *44*). The purple endosymbionts can presumably survive under low oxygen concentrations as they retained superoxide dismutases for oxygen radical detoxification (data S1) and have micro-aerobic respiratory chains with a cytochrome *cbb*_3_ (Fig. 3) that can scavenge oxygen (*45*). This agrees with the fact that *P. tenue* is found in the hypoxic-to-oxic transition zones of muddy ponds.

At least two other physiological modes are allowed by the metabolic complexity of the *P. tenue* symbiotic consortium (see Fig. 4C). Moreover, intermediate metabolic states between these four major modes might also occur in transitional environments. For example, the green algal symbiont might perform some oxygenic photosynthesis (*46*) or a form of anoxygenic photosynthesis through cyclic electron flow under low-light and anaerobic conditions (*47*), and the purple symbionts could express their photosynthetic chain alongside its micro-aerobic respiratory chain (*48*). The accumulation of starch granules around pyrenoids in chloroplasts supports the notion that the green algae can photosynthesize oxygenically under some conditions (see fig. S2C and Supplementary Text). The *P. tenue* consortium is thus endowed with great physiological, and potentially also ecological, flexibility. This flexibility might allow *P. tenue* to temporarily survive under suboptimal conditions (fig. S9) such as oxygenated waters; *P. tenue* survives for a few days but does not divide and eventually disappears in laboratory samples exposed to atmospheric oxygen. Given the complex physiology of each symbiotic partner, sequencing single-cell metatranscriptomes from this unusual symbiotic consortium under different environmental conditions (e.g., light-anoxia versus dark-oxia) is necessary. These future efforts will test the physiological hypotheses presented here and provide more certainty about the metabolic interactions between *P. tenue* and its symbionts.

### The symbiotic merger of disparate physiologies created an unusual ecological niche

By studying a unique purple-green ciliate, we found the first example of a photosynthetic purple “sulfur” bacterium (Chromatiaceae, Gammaproteobacteria) with an endosymbiotic lifestyle. This anaerobic purple bacterium shares its host cell with an oxygenic green alga (*Chlorella* sp.). This puzzling coexistence appears to be possible because of the physiological versatility offered by each symbiotic partner and the reductive specialization of the purple bacterial endosymbiont. For example, the purple bacterium lost sulfur dissimilation genes, and both symbionts and host have the genes for respiring micro-aerobically and fermenting anaerobically. Moreover, the evolutionary merging of two distinct photosymbionts and a facultatively anaerobic predator has created a symbiotic consortium with a complex set of physiological properties not shared by any other species known. The niche of this symbiotic consortium, however, is not simply the union of the niches of each symbiont but presumably encompasses most of the ciliate host and purple symbiont niches and partially overlaps with that of the green symbiont (Fig. 4C and fig. S9). The outcome is a eukaryote that actively swims in hypoxic sediments and combines phagotrophy with anoxygenic photosynthesis. *P. tenue* thus represents an extraordinary example of how symbiosis merges disparate physiologies and allows emergent consortia to create novel ecological niches.

## MATERIALS AND METHODS

### Sampling and storage of live material

Water and sediment samples were taken from pond II [naming according to (*15*)] of the Simmelried peat bog area near Hegne, Germany (47.717767, 9.09375) at a depth of about 30 cm. These samples were transported in 200-ml jars and lastly combined into 1-liter bottles with a loose screw cap to allow the formation of a bottom sediment and different zones. These bottles were kept at 15°C under dim, indirect light with a 14:10-hour light/dark cycle.

### DNA extraction from single cells and PCR amplification

Individual cells visually identified as *P. tenue* were isolated from diluted sediment of the 1-liter bottles using glass micropipettes, washed twice in sterile distilled water, and transferred into a maximum of 10 μl of nuclease-free water in a 0.2-ml tube. These tubes were then snap-frozen in liquid nitrogen and stored at −20°C. To extract genomic DNA (gDNA) from isolated cells, the following components were added to the frozen tubes in order: 50 μl of tris-HCl buffer (10 mM; pH 8.5), 50 μl of Chelex 100 (5%; Sigma-Aldrich), and 2 μl of proteinase K (20 mg/ml; Thermo Fisher Scientific). The solution was then mixed by vortexing for 15 s, briefly centrifuged, and then incubated at 56°C for 45 min and lastly at 98°C for 20 min. After incubation, the samples were centrifuged at 17,000*g* for 3 min and stored at 4°C. PCR of single-cell gDNA extract was performed using Q5 DNA Polymerase (New England Biolabs) following the manufacturer’s protocol. A volume of 4 μl of gDNA extract was used per 50-μl PCR reaction mix. To amplify most of the rRNA gene operon (comprising the 18*S* rRNA gene, ITS1-5.8*S* rRNA-ITS2, and the D1D2 region of the 28*S* rRNA gene) of the ciliate host, the primers Euk_A (5′-AAC CTG GTT GAT CCT GCC AG-3′) and D1D2rev2 (5′-ACG ATC GAT TTG CAC GTC AG-3′) were used (*49*, *50*). The PCR cycle was as follows: 98°C for 30 s (1×), 98°C for 10 s, 66°C for 15 s, 72°C for 70 s (35×), and 72°C for 120 s (1×). To amplify the *rbcL* gene from the green algal endosymbiont, two successive rounds of PCR (semi-nested) were done using the primers MaGo1F (5′-ATG TCA CCA CAA ACN GAA AC-3′) and MaGo3R (5′-GTA TCR ATH GTW TCA AAT TC-3′) for the first PCR and MaGo2F (5′-ATG TCA CCA CAA ACN GAA ACT AAA GCW GG-3′) and MaGo3R (5′-GTA TCR ATH GTW TCA AAT TC-3′) for the second PCR (*51*). The first PCR protocol was as follows: initial denaturation (98°C, 30 s); 30 cycles of denaturation (98°C, 10 s), annealing (50°C, 15 s), and elongation (72°C, 45 s); followed by final elongation (72°C, 120 s). The second PCR was done with the same protocol except that 35 cycles were used. To amplify the 16*S* rRNA gene from the bacterial endosymbiont, the primers 16S_Bact_8F (5′-AGA GTT TGA TCC TGG CTC AG-3′) and SSU_Univ_1542R (5′-AAG GAG GTG ATC CAG CCG CA-3′) were used (*52*). The PCR protocol was as follows: initial denaturation (98°C, 30 s); 35 cycles of denaturation (98°C, 10 s), annealing (65°C, 15 s), and elongation (72°C, 45 s); followed by final elongation (72°C, 120 s). PCR amplicons were purified with the NucleoSpin Gel and PCR Clean-up Kit (Takara Bio) and sequenced with the Sanger sequencing method (Eurofins Genomics, Ebersberg, Germany) using the primers 18S_1400F (5′-CTG CCC TTT GTA CAC ACC GCC CGT C-3′), D1D2-rev2 (5′-ACG ATC GAT TTG CAC GTC AG-3′), and those mentioned above.

### Phylogenetic analyses

Sanger reads were assembled on Benchling (https://benchling.com). Overlapping consensus sequences of the ciliate rRNA operon were assembled in Geneious (www.geneious.com). Representative sequences for the Heterotrichea (ciliate rRNA gene operon) were retrieved from Shazib *et al.* (*19*). Representative sequences for the Chlorellaceae (*rbcL*) were retrieved from a BLASTn search against the National Center for Biotechnology Information (NCBI) GenBank nr database. Representative sequences for the Chromatiaceae and Sedimenticolaceae (16*S* rRNA gene) were retrieved from the SILVA rRNA database (*53*). The *COI* and *rbcL* genes were aligned by codons with the MAFFT algorithm in TranslatorX (*54*). The 16*S*, 18*S*, 5.8*S*, D1D2-28*S* rRNA genes, and the ITS1 and ITS2 regions were aligned separately with MAFFT v7.310 (*55*) using the G-INS-I method and then trimmed with Trimal v1.4.rev15 (*56*) and the -automated1 option. The final selection of taxa was performed visually with the aim of maximizing phylogenetic diversity. The 18*S*, 5.8*S*, D1D2-28*S* rRNA genes, and the ITS1 and ITS2 regions of the ciliate rRNA operon were analyzed as separate partitions. Phylogenetic trees were inferred using IQ-TREE v.2.0.3 (*57*) and the best-fit model according to Bayesian information criterion as implemented in ModelFinder (*58*).

### FISH and fluorescence microscopy

Using a 16*S* rRNA gene sequence obtained by Sanger sequencing that showed >98% identity with *Thiodictyon* species (Chromatiaceae, Gammaproteobacteria), we retrieved potential FISH probes with the “Design Probes” web tool of the software toolset DECIPHER under default hybridization conditions (*59*). One of the probe sequences with maximum specificity (score of 0), reasonable hybridization efficiency, and no reported cross-hybridizations with nontarget sequences was analyzed with the online tool “mathFISH” (*60*) and manually modified to optimize binding to the target sequence. The resulting probe Thio643 (5′-CTC TCA GAC TCT AGC GCG TCA-3′) was lastly checked for specificity in silico with the “probeMatch” database (*61*) and the SILVA probe match and evaluation tool “TestProbe 3.0.” The probe showed no hits with zero mismatches in both databases and only few hits with one mismatch (18 hits in probeMatch: “unclassified Chromatiaceae”; 4 hits in TestProbe: uncultured Gammaproteobacteria) indicating high specificity (as of March 2020). The Cy3-conjugated probe Thio643-Cy3 was ordered (biomers.net GmbH, Ulm, Germany), and its binding to bacterial cells from *P. tenue* was evaluated under varying formamide concentrations (0 to 60%) using a modification of previously published FISH protocols (*62*, *63*). In brief, cells of *P. tenue* were isolated from natural samples, washed twice in distilled water, placed on gelatin-coated glass slides (coated with 0.1% gelatin in water), and air-dried. Some ciliate cells were deliberately burst with a pipette tip to spread the endosymbionts. Cells on dry slides were fixed with formaldehyde (3.7% in water) for 10 min, then rinsed in distilled water, and air-dried. To minimize potential autofluorescence, photosynthetic pigments of bacteria and algae were first extracted from the samples by incubating the slides in a methanol:water mixture (8:2) at 65°C for 10 min, then rinsed in ethanol (100%), and air-dried. The cells were then covered with 50 to 100 μl of hybridization buffer containing probe Thio643-Cy3 at an appropriate concentration (5 ng/liter) and 0 to 60% formamide [according to (*63*)] and then incubated in a moist chamber at 46°C for 90 min. After incubation, the slides were quickly rinsed with warm washing buffer (48°C) and subsequently incubated in washing buffer at 48°C for 25 min. Last, slides were rinsed with distilled water, air-dried, and mounted with SlowFade Diamond Antifade Mountant (Thermo Fisher Scientific).

Routine epifluorescence microscopy of FISH samples was done with the Zeiss Axiophot microscope equipped with phase contrast optics, the Zeiss filter set 43 HE (excitation, 550/25 nm; emission, 605/70 nm), and the camera AxioCam HRc (Carl Zeiss). For confocal laser scanning microscopy, a Leica TCS SPE system and the Leica Confocal Software (Leica Microsystems) were used. The samples were excited with lasers (561 nm for Cy3 and 488 nm for chlorophyll) and scanned in three dimensions with step sizes of <1 μm. Confocal laser scanning microscopy (CLSM) stacks were processed and analyzed with the software LAS AF lite (Leica Microsystems) and the image processing package Fiji (*64*). This included the selection of pseudocolors for fluorescence channels, the adjustment of brightness, and the combination of different channels (merged channels) and/or of several focal planes (Z-projections).

### Transmitted light microscopy

For high-resolution imaging of live cells, two microscopes were used: an Olympus BX 50 microscope and a Zeiss IM35 inverted microscope, both equipped with differential interference contrast optics, high-resolution objective lenses, and an electronic flash. Digital images were taken with an Olympus E-P5 digital camera and Canon EOS 6D digital single lens reflex camera, respectively, and processed with Adobe Photoshop CS4 (Adobe Systems).

### Transmission electron microscopy

About 30 cells of *P. tenue* were isolated from natural samples and collected in about 200 μl of distilled water. This cell suspension was quickly injected into 200 μl of freshly prepared fixative [80 μl of 25% glutaraldehyde, 200 μl of 4% osmium tetroxide solution, and 120 μl of half-strength medium Waris-H (pH 7) with 1.5 mM Hepes], resulting in a final concentration of 2.5% glutaraldehyde and 1% osmium tetroxide. Cells were incubated for 1 min at room temperature and for another 45 min at 6°C, then quickly washed with distilled water (twice, supernatant was removed after cells settled), and dehydrated by a series of acetone:water mixtures (30, 50, 80, and 90%) and pure acetone (twice) for 5 min each at −20°C. Acetone was then replaced by 50% EPON in acetone, incubated for 24 hours at about 6°C for infiltration, and then the tube was opened and stored for another 24 hours in a fume hood to let the acetone evaporate. The sample was then mixed with fresh EPON and cured at 65°C for 1.5 days. Single ciliate cells were sectioned (~60 nm thickness) with a Leica EM UC7 ultramicrotome (Leica Microsystems), stained with uranyl acetate (1%, 15 min) and Reynolds’ lead citrate solution (3.5 min), and imaged with a CM10 TEM (FEI Europe Main Office, Eindhoven, The Netherlands) and a Gatan ORIUS SC200W TEM charge-coupled device (CCD) camera (Gatan Inc., Pleasanton, CA).

### Hyperspectral imaging

Several cells of *P. tenue* were picked from a natural sample, washed in demineralized water, placed onto a coverslip, and let air-dry. The dry cells were then covered by immersion oil and imaged with a CytoViva Hyperspectral Microscope (CytoViva Inc., AL, USA) in the visible and near-infrared range (400 to 1000 nm) using the Olympus UPlanFL N 100×/1.30 oil objective (Olympus Europa SE & Co. KG, Hamburg, Germany). The microscope was based on an Olympus BX43 stand equipped with a SPECIM spectrograph (Specim, Spectral Imaging Ltd., Oulu, Finland) and the pco.pixelfly USB CCD Camera (PCO AG, Kelheim, Germany). The sample was illuminated with transmitted light of a Fiber-Lite DC-950 halogen illuminator (Dolan-Jenner Industries) coupled into the Olympus BX43 stand. Hyperspectral images were recorded and analyzed with the ENVI software (CytoViva Inc., AL, USA). Per sample type (purple bacteria and green algae), the recorded transmission spectra of four rectangular areas (defined as regions of interest; see fig. S4A) were averaged and used for analysis. First, the averaged transmittance spectra were normalized to the recorded lamp spectrum (also obtained by four averaged measurements) and then converted to relative absorbance spectra for each sample type. The exact sampling areas and absolute absorbance spectra are shown in fig. S4A.

### Single-cell whole-genome amplification and sequencing

Individual ciliates isolated, washed, and deposited in 0.2-ml tubes as detailed above were inspected under low magnification to confirm cell integrity and then snap-frozen in liquid nitrogen. The REPLI-g Advanced DNA Single Cell Kit (QIAGEN) was used to perform a single-cell whole-genome amplification (WGA) on three individual ciliates. Three different variations of the WGA protocol were performed: Variation 1 was applied on single cells and followed the default protocol. Variation 2 introduced a 65°C incubation step (10 min) to facilitate lysis of endosymbionts. Variation 3 followed the default protocol on a single-cell DNA extract obtained with Chelex 100 (see above for details). Illumina sequencing libraries were made with the Illumina TruSeq kit from the products of all three WGA variations. Illumina short-read sequencing (2 × 150 bp) was done on a NovaSeq Illumina sequencing platform at the Cologne Center for Genomics at the University of Cologne, Germany.

### Metagenome assembly and analysis

Raw Illumina short reads were quality assessed and filtered, and adapters were trimmed with afterqc v.0.9.6 (-f 8 -t 2) (*65*). Coassembly of multiple sequenced libraries was done with SPAdes v.3.14.0 (--sc) (*66*). Scaffolds were then processed using Anvi’o v.6.1 (*67*) and the metagenomic pipeline. In brief, all scaffolds shorter than 1000 bp were discarded, and reads from each library were separately mapped on the scaffolds to obtain their coverage values. Taxonomic affiliations for each contig were assigned on the basis of a consensus of best DIAMOND (*68*) hits for each scaffold gene against the NCBI nr database as of October 2019. Scaffolds were organized according to the composition and coverage and manually visualized and inspected with anvi-interactive (*67*). The scaffolds in the simple metagenome clearly fell into three main clusters and closely followed the taxonomy of each of the three symbiotic partners in *P. tenue* as determined with PCR/Sanger sequencing. The scaffold cluster (bin) annotated as *Thiodictyon* was further manually refined with anvi-refine (*67*) by relying on composition, coverage, taxonomic, and completeness/redundancy criteria. The metagenome-assembled genome for “*Ca.* T. intracellulare” was estimated to be 99.16% complete and 0.74% redundant (a measure of potential contamination) by CheckM v.1.0.18 (*69*) and the taxonomic workflow for the Gammaproteobacteria. Scaffold clusters that taxonomically corresponded to the *P. tenue* ciliate host and the endosymbiotic green alga *Chlorella* sp. were identified manually through Anvi’o and estimated to be 93.0 and 77.2% complete by BUSCO v.4.0.6 (*70*) based on the alveolata_odb10 and chlorophyta_odb10 lineage datasets, respectively.

### Mitochondrial genome (mitochondrial DNA) annotation

Mitochondrial genome scaffolds were identified by BLASTp searches of mitochondrial DNA (mtDNA) proteins (AN) from *Gruberia lanceolata* (Heterotrichea, Ciliophora) (*71*) against the *P. tenue* metagenome. The scaffolds identified were then inspected through anvi-interactive, and they were found to cluster together according to their composition. Attempts to circularize and/or extend the scaffolds were done with NOVOPlasty v.3.8.2 (*72*). Several NOVOPlasty iterations were performed with different seeds. The resulting contigs were assembled in Geneious to resolve the scaffold organization manually. A preliminary annotation was carried out with MFannot (https://github.com/BFL-lab/Mfannot). Because of the extreme divergence of ciliate mtDNA protein-coding genes, all predicted open reading frames (ORFs) larger than 50 amino acids were searched against the NCBI GenBank nr database and a ciliate mtDNA database (containing all conserved ORFs of unknown function). In addition, pHMM searches against the Pfam database were performed.

### Inference of the anaerobic energy metabolism of the *P. tenue* ciliate host and its green endosymbiont *Chlorella* sp.

A set of query sequences reported for anaerobic enzymes across eukaryotes in (*73*, *74*) were retrieved and used for BLASTp, BLASTn, and Exonerate (*75*) searches against the draft genome of the *P. tenue* ciliate host. Similarly, a set of protein query sequences for enzymes annotated as involved in anaerobic energy metabolism and fermentative pathways in *C. reinhardtii* (*29*, *30*) were manually retrieved from UniProtKB. These were used to search the draft genome of the endosymbiotic *Chlorella* sp. using Exonerate and the options “--refine full --model protein2genome.” Best BLAST and Exonerate hits were then searched against the nonredundant (nr) dataset to confirm their presumed homology.

### Inference of the metabolism of the purple endosymbiont *Ca.* T. intracellulare

The proteome of “*Ca.* T. intracellulare” was inferred with Prodigal v.2.6.3 (-p single) (*76*). Annotation of the proteome was done with InterProScan v.5.41-78.0, BlastKOALA, and KofamKOALA at the Kyoto Encyclopedia of Genes and Genomes server (*77*). Reciprocal BLAST searches against the predicted proteome of “*Ca.* T. intracellulare” were performed using query sequences for genes involved in sulfur metabolism described and annotated in (*27*). Reciprocal BLAST searches against the predicted proteome of “*Ca.* T. intracellulare” using the query sequences for metabolic genes present in the genomes of “*Ca.* T. syntrophicum” and *A. vinosum* DSM-180, as discussed in (*25*, *28*), were also performed. Moreover, additional reciprocal BLAST searches were performed for additional genes of interest not discussed in (*25*, *28*). Rough predictions of pseudogenes in the genomes of “*Ca.* T. syntrophicum” and *Ca.* T. intracellulare were performed with PGAP (*78*) and DFAST (*79*). A more exhaustive prediction of pseudogenes in the genome of “*Ca.* T. syntrophicum” was performed with Pseudofinder (*80*).

## SUPPLEMENTARY MATERIALS

Supplementary material for this article is available at http://advances.sciencemag.org/cgi/content/full/7/24/eabg4102/DC1
